# Rapid and spontaneous resolution of hemorrhagic macular hole retinal detachment and subretinal hemorrhages in an eye with pathologic myopia: a case report

**DOI:** 10.1186/s12886-020-01653-0

**Published:** 2020-09-29

**Authors:** Taiju Ito, Tae Igarashi-Yokoi, Kosei Shinohara, Takeshi Yoshida, Kyoko Ohno-Matsui

**Affiliations:** 1 Department of Ophthalmology and Visual Science, Graduate School of Medical and Dental Sciences, Tokyo Medical and Dental University, 1-5-45 Yushima, Bunkyo-ku, Tokyo, 113-8519 Japan; 2 Tokyo Metropolitan Komagome Hospital, 3-18-22 Honkomagome, Bunkyo-ku, Tokyo, 113-8677 Japan; 3 Japanese Red Cross Musashino Hospital, 1-26-1 Kyonancho Musashino-shi, Tokyo, 180-0023 Japan

**Keywords:** Pathologic myopia, Macular hole retinal detachment, Choroidal neovascularization, Punctate inner choroidopathy, Multifocal choroiditis

## Abstract

**Background:**

To report a rare case of pathologic myopia in which a choroidal neovascularization (CNV) induced a hemorrhagic macular hole retinal detachment (MHRD), and then both the CNV and MHRD disappeared simultaneously in 5 days.

**Case presentation:**

A 76-year-old man with pathologic myopia complained of distorted vision in his left eye of 1-week duration. The visual acuity in the left eye was 20/20 and the axial length was 31.0 mm. Ophthalmoscopic examinations of the left eye showed many retinal hemorrhages and whitish lesions on a background of severe diffuse myopic atrophy. Swept-source OCT (SS-OCT) showed multiple hyperreflective vertical finger-like projections extending into the outer retina that corresponded to the area of the botryoidal-shaped retinal hemorrhages. The SS-OCT images also showed many subretinal infiltrations adjacent to linear retinal hemorrhages with a disruption of the adjacent ellipsoid zone of the photoreceptors. Fluorescein angiography (FA) showed early hyperfluorescence and late leakages corresponding to the areas of the hemorrhages or adjacent to the linear retinal hemorrhages. These results suggested that the development of the inflammatory CNV was related to the outer retinopathy or choroiditis as in eyes with punctate inner choroidopathy or multifocal choroiditis rather than myopic CNV. We planned an intravitreal anti-vascular endothelial growth factor (anti-VEGF) injection but the patient noticed a sudden reduction of the visual acuity a few days before the anti-VEGF injection. The left fundus showed a MHRD due to the subretinal hemorrhage. Five days later, the SS-OCT images confirmed a recession of the CNV and a resolution of the MHRD.

**Conclusions:**

Rapid and spontaneous resolution of both myopic CNV and hemorrhagic MHRD suggest that there may have been a mutual mechanism causing the MHRD and CNV. A careful follow-up before doing surgery may be a choice for hemorrhagic MHRD in eyes with pathologic myopia.

## Background

A macular hole retinal detachment (MHRD) is a serious retinal disorder that can progress to blindness, and it affects 3.4 to 4.7% of patients with pathologic myopia [1–6]. The pathogenesis of a MHRD is related to the abnormal characteristics of pathological myopic eyes such as deep posterior staphylomas, severe myopic chorioretinal atrophy, epiretinal membrane, vitreal traction, retinoschisis, and posterior vitreous schisis. Complex mechanical interactions of these factors are involved in the development of the MHRDs. Vitreous surgery is usually performed on eyes with a MHRD, however there are at least four reports on a spontaneous resolution of the MHRD in highly myopic eyes [3–6].

Outer retinopathy and choroiditis, such as punctate inner choroidopathy (PIC) and multifocal choroiditis (MFC), are rare disorders which are included in the white dot syndrome spectrum, and they mostly occur in myopic patients [7, 8]. Most researchers believe that PIC and MFC are the same underlying disease entity based on the ocular findings detected by recent multimodal imaging [7, 8]. Inflammatory choroidal neovascularization (CNV), one of the major complications of outer retinopathy and choroiditis, is reported to be present in 30 to 75% of eyes of patients with PIC or MFC [9]. The inflammatory CNV is difficult to distinguish from myopic CNV [7, 8]. However, a prompt diagnosis of inflammatory CNV is critical for preserving vision because its management may require different therapeutic procedures.

We present our findings of a rare case of pathologic myopia in which a CNV was associated with a hemorrhagic MHRD. To the best of our knowledge, this is the first report of hemorrhagic MHRD that developed secondary to CNV in eyes with pathologic myopia. Unexpectedly, the activity of the CNV and MHRD disappeared simultaneously in 5 days without treatment. The mechanism controlling the rapid recession of both hemorrhagic MHRD and CNV will be discussed.

## Case presentation

A 76-year-old man with pathologic myopia who had received an anti-vascular endothelial growth factor (anti-VEGF) injection for myopic CNV in the right eye a year earlier was studied. His ocular history also included prior cataract surgery in both eyes and an inner lamellar macular hole in the left eye. He was not taking any medications and had no other medical diseases.

**Fig. 1:**
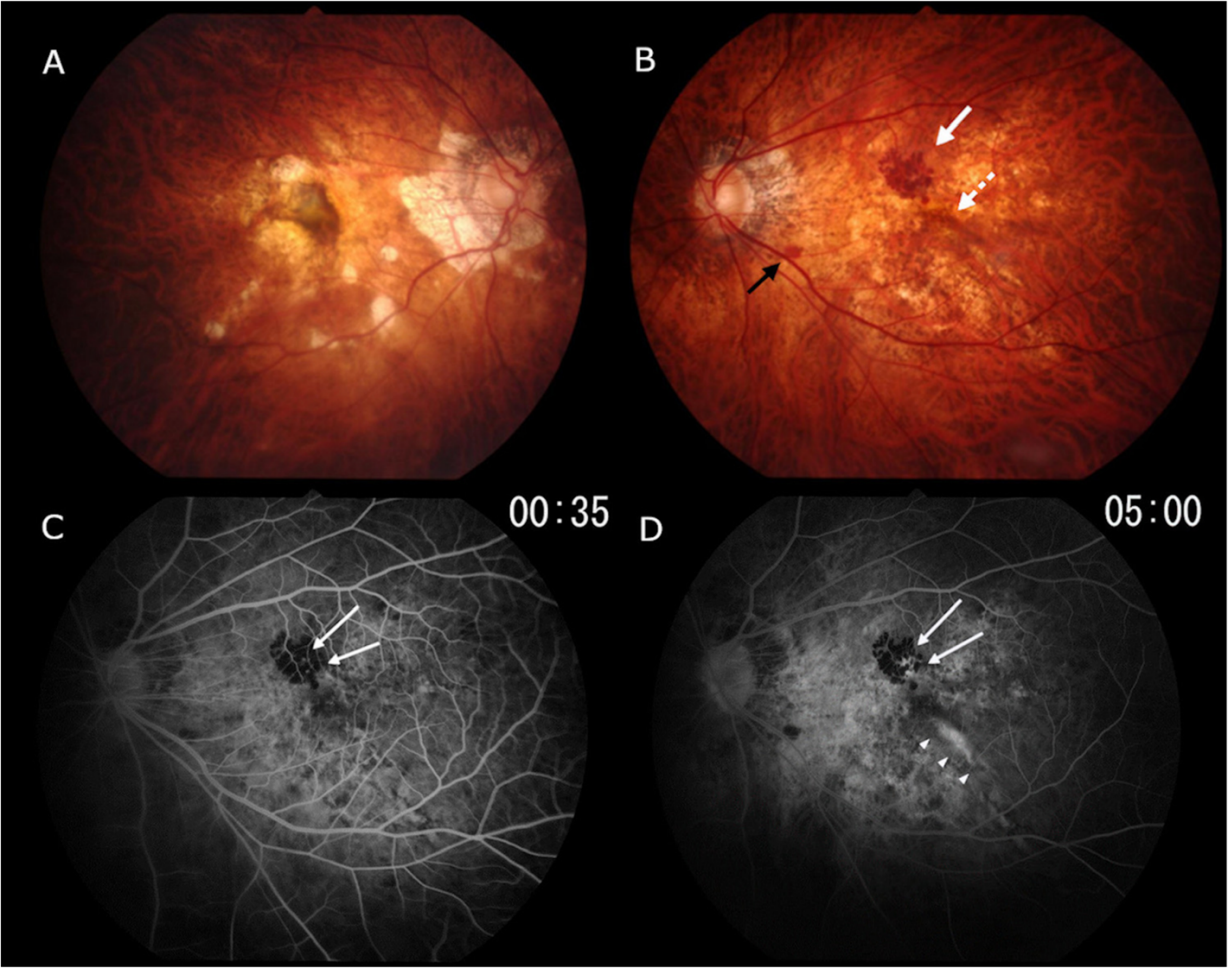
Color fundus photographs and fluorescein angiograms at the onset of choroidal neovascularization (CNV). **a**: Color fundus photograph of the right eye shows many areas of patchy atrophies and Fuchs’ spots on a background of severe diffuse atrophy. **b**: Left eye shows many whitish lesions and retinal and subretinal hemorrhages. The hemorrhages on the superonasal side of the fovea are relatively large and botryoid-shaped (white arrow), and the hemorrhages in the parafoveal region are linear (dotted arrow). The hemorrhages on the inferotemporal side of the optic disc are small, flat, and round (black arrows). **c**: Early phase fluorescein angiogram showing that the areas of the retinal and subretinal hemorrhages are hypofluorescent at the early phase. There are also early hyperfluorescence within the area of the botryoidal-shaped hemorrhages (arrows). **d**: Late phase of fluorescein angiogram. During the entire phase, the areas corresponding to the retinal and subretinal hemorrhages are hypofluorescent due to blockage. It can also be seen that the late leakages within the area of the botryoidal-shaped hemorrhages (arrows) and adjacent to the linear retinal hemorrhages (arrowheads), which are suspected to be multiple developments of the CNV

**Fig. 2:**
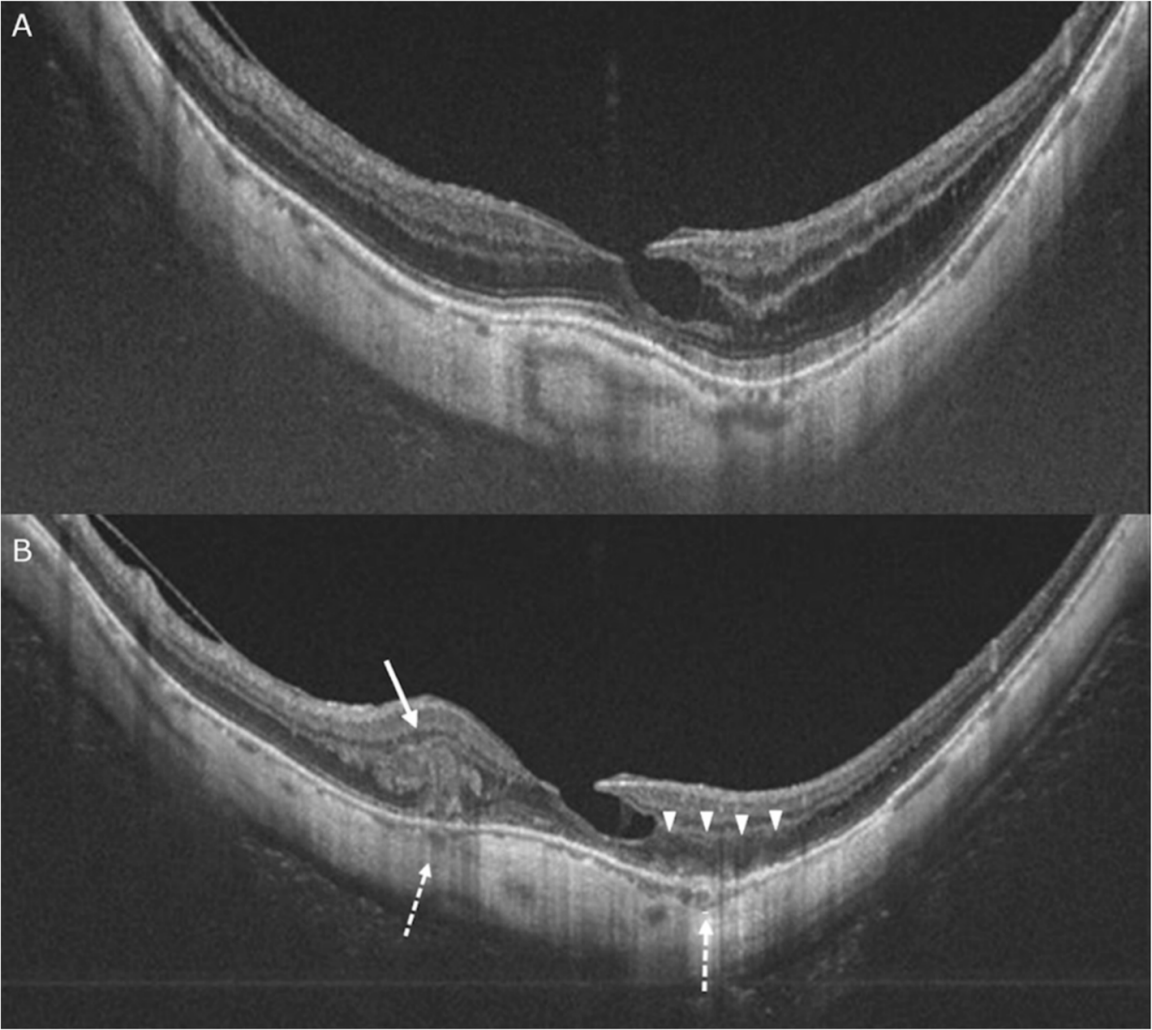
Swept-source OCT (SS-OCT) images of the left eye before and at the onset of choroidal neovascularization (CNV). **a**: SS-OCT image before the symptoms occurred in the left eye. Initially, an inner lamellar macular hole was detected. No abnormalities were observed in the ellipsoid zone except in the area of the MH. **b**: SS-OCT image of the left eye shows multiple, distinctive, vertical finger-like projections extending into the outer retina, a pitchfork sign, corresponding to the area of the botryoidal-shaped hemorrhage (white arrow) and many subretinal infiltrations adjacent to the linear retinal hemorrhaged (arrowheads) at the site of the CNV. In the areas of these retinal infiltrations, the choroid is thickened (dotted arrows). On the temporal side of the fovea, the ellipsoid zone is extensively disrupted

He returned to our clinic with a complaint of distorted vision in the left eye which he first noted one week prior to our examination. His visual acuity was 20/40 in the right eye and 20/20 in the left eye, and the intraocular pressure was 17 mmHg in the right eye and 15 mmHg in the left eye. The refractive error (spherical equivalent) was − 0.38 diopters (D) in the right eye and − 0.50 D in the left eye. The axial length was 30.9 mm in the right eye and 31.0 mm in the left eye. Slit-lamp examination was unremarkable. No inflammatory cells were detected in the anterior chamber or posterior vitreous. Dilated ophthalmoscopic examinations of the right eye showed multiple patchy chorioretinal atrophies and myopic CNV at the scar phase on a background of severe diffuse chorioretinal atrophy (Fig. 1a). The left eye showed 3 sites of retinal and subretinal hemorrhages and many whitish lesions on a background of severe diffuse chorioretinal atrophy (Fig. 1b). The hemorrhages were seen in the superior-nasal area of the central fovea and were relatively large and botryoidal-shaped (Fig. 1b: white arrow). The ones in the parafoveal region were linear and small (Fig. 1b: dotted white arrow), and those in the inferotemporal side of the optic disc were round (Fig. 1b: black arrow). The swept-source OCT (SS-OCT) images of the left eye showed disruption of the inner segment/outer segment junction with hyperreflective anterior projections into the outer retina that corresponded to the area of the botryoidal-shaped hemorrhages (Fig. 2b: arrow). These SS-OCT images also showed subretinal infiltrations adjacent to the linear retinal hemorrhages (Fig. 2b: arrowheads) and a disruption of the adjacent ellipsoid zone (Fig. 2b: dotted arrows). The quality of the OCTA images was poor due to unstable fixation, excessively long axial length, and severe myopic chorioretinal atrophy. The fluorescein angiographic (FA) images showed that the retinal and subretinal hemorrhages were hypofluorescent due to blockage (Fig. 1c, d). The FA images also showed early hyperfluorescence and late leakages corresponding to the area of the botryoidal-shaped hemorrhages (Fig. 1d: arrows) and also adjacent to the linear retinal hemorrhages (Fig. 1d: arrowhead). These sites were suspected to be the area where the Type 2 CNV developed.

**Fig. 3:**
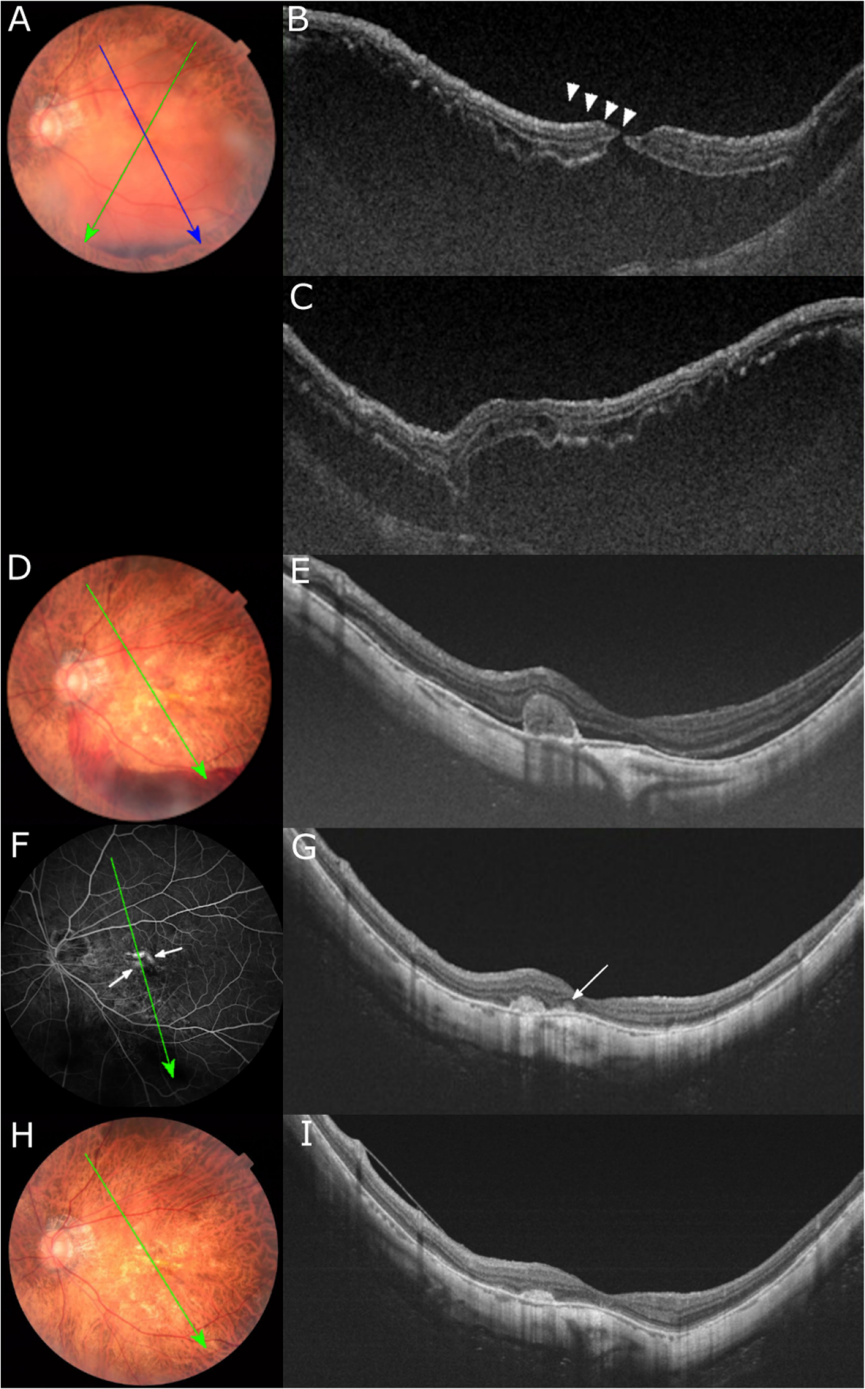
Color fundus photographs and swept-source OCT (SS-OCT) image after the development of macular hole retinal detachment (MHRD). **a**: Color fundus photograph at the onset of the MHRD, and a few days before the anti-vascular growth factor drug injection for the choroidal neovascularization (CNV). A newly developed MHRD due to the subretinal hemorrhage can be seen. **b**: SS-OCT image corresponding to the green oblique line in Fig. A shows a small MH of 80 μm diameter, and there is a hyperreflective line (arrowheads) most likely the residual posterior vitreous membrane covering the MH. **c**: Another SS-OCT section crossing the area of the CNV under the previous botryoidal-shaped hemorrhage at the superonasal side of the central fovea corresponding to the blue oblique line in Fig. A. The adhesion between the CNV and the outer retinal layer seemed to prevent the retinal detachment from proceeding upward. **d**: Color fundus photograph on the 5th day after the development of MHRD. The subretinal fluid is essentially absent. New subretinal hemorrhages are still present. **e**: Preoperative SS-OCT image confirms the spontaneous resolution of the MHRD. In addition, the CNV has regressed with enclosure by the retinal pigment epithelium. F: Fluorescein angiography (FA) image four months after the spontaneous resolution of the MHRD shows late leakages corresponding to the area of the recurrent CNV (arrows). The dye leakage observed adjacent to the linear retinal hemorrhages in Fig. 1d has completely disappeared. G: SS-OCT image shows the subretinal hyperreflective infiltration (arrow) adjacent to the regressed CNV. **h**: and **i**: Color fundus photograph and SS-OCT image 8 month after the spontaneous resolution of MHRD. Subretinal hemorrhage completely absent and vision improved to 20/25

We planned anti-VEGF drug injection to treat the developing CNV but the patient noticed a sudden reduction of his visual acuity in the left eye 3 weeks later. We re-examined the patient and found that a MHRD had developed in the left fundus with subretinal hemorrhages (Fig. 3a). The SS-OCT images showed a MH with a diameter of 80 μm, and a hyperreflective line which was suspected to be the residual posterior vitreous membrane (Fig. 3b). We planned a vitrectomy 5 days later, however the preoperative SS-OCT images showed a closed MHRD (Fig. 3e) and the myopic CNV had regressed (Fig. 3e). Four months after the spontaneous closure of the MHRD, the CNV recurred (Figs. 3f, g). The dye leakage observed adjacent to the linear retinal hemorrhages in Fig. 1d had completely disappeared. Anti-VEGF drug injection was performed to treat the recurred CNV. The visual acuity in the left eye improved to 20/25 from 20/200.

## Discussion and conclusions

We studied a rare case of pathologic myopia with an active CNV and a hemorrhagic MHRD which was followed by a rapid resolution of the MHRD and CNV without any treatment. The acute development and rapid resolution of both the MHRD and CNV in 5 days suggest a common mechanism probably caused these two pathologies. The multiple retinal hemorrhages in this case need to be differentiated from the simple macular hemorrhage, lacquer cracks, myopic CNV, and inflammatory CNV related to outer retinopathy or choroiditis. The botryoidal-shaped hemorrhages were present in the superior-nasal area of ​​the central fovea (Fig. 1b: white arrow), and the OCT images showed subretinal hemorrhage with projections along Henle’s layer (Fig. 2b). The CNV was not obvious in this image. Based solely on this OCT image, it appeared as if the subretinal bleeding caused by the formation of a lacquer crack was the cause. However, in the fluorescein angiograms (Figs. 1c, d), two dots of hyper-fluorescence were seen in the early phase which enlarged in the late phase. Thus, these two hyper-fluorescent images showed ‘dye leakage’ because the area of hyper-fluorescence enlarged with time. In contrast, there was no hyper-fluorescent area of the simple macular hemorrhages due to new lacquer crack formation. In addition, a staining of the subretinal tissue was observed in the OCT image and late phase fluorescein angiogram after the hemorrhage and the MHRD were completely resolved (Fig. 3e, f, g). In the case of simple macular hemorrhage, lacquer cracks are observed in the area of previous hemorrhage. However, these OCT images and fluorescein angiograms are distinctly different from lacquer cracks and more suggestive of CNV. The linear retinal hemorrhages in the parafoveal region (Fig. 1b: dotted arrow) and the fluorescein angiograms (Figs. 1c, d) showed dye leakage. However, four months after the spontaneous resolution of the MHRD (Fig. 3f), the dye leakage observed had entirely disappeared (Fig. 1d). Because a relatively large myopic CNV is generally unlikely to disappear completely, we must consider some kind of inflammatory lesion without CNV, although inflammatory CNV cannot be completely ruled out.

Generally, it is very difficult to differentiate it from a myopic CNV especially when the inflammatory CNV is not accompanied by cells in the vitreous [7, 8]. However, the significant characteristics of inflammatory lesions associated with outer retinopathy and choroiditis can be used to differentiate these inflammatory CNV from myopic CNV and other myopic choroidal lesions [7, 8, 10]. The published findings in inflammatory lesions in the high-resolution OCT images include elevations of the sub-RPE, dehiscence of the RPE, subretinal infiltration, attenuation or absence of the ellipsoid zone, and deeper penetration of the OCT signal [7]. Additionally, multiple distinctive vertical finger-like projections that extend from the area of active CNV into the outer retina are reported to be specific findings of inflammatory CNV related to PICs and MFCs [8]. The distinctive mushroom-like hyper-reflections on the OCT at site of the botryoidal-shaped hemorrhages observed in our case were similar to such sign reported in inflammatory CNV related to PICs and MFCs. However, in our case, there was a pre-existing retinoschisis at the same site before the bleeding, and it was possible that the unique reflective mass on the OCT resulted from exudation of bleeding at the retinoschisis site. Even so, the atypical fundus features in the high-resolution OCT images, such as multiple, recurrent CNV with pitchfork-like appearance in the OCT images or a wide area of ellipsoid zone absence, suggested that the CNV in our case was inflammatory rather than due to pathologic myopia.

Because of his advanced age and good vision, and anticipating the anti-inflammatory effect of anti-VEGF drugs, we did not consider oral steroids at this time. As best we know, there are no reports that describe the changes that accompany inflammatory CNV that lead to the development of a MH or a MHRD. Because the hemorrhagic MHRD occurred immediately after the development of the inflammatory CNV, the exudative changes caused by the inflammation of the outer retina may have been the trigger for the MHRD. There was a pre-existing inner laminar macular hole and then inflammatory CNV with relatively massive hemorrhage that developed diagonally across the central fovea on a background of severe diffuse atrophy. These changes might have accelerated the damage of the foveal structures which was already fragile due to the inner laminar macular hole, leading to the formation of the MH and finally progressing to the MHRD.

Interestingly, the retinal reattachment in our case occurred within 5 days together with a rapid resolution and shrinkage of the CNV without any treatment. Earlier studies reported a spontaneous resolution of a MHRD without active CNV (Table 1) [3–6], however it required a much longer time of one month to 4 years [4–6]. As a possible mechanism for the rapid and spontaneous resolution of both MHRD and active CNV, the presence of subretinal blood and vitreous may be considered. Several studies have reported that the application of autologous adjuvant blood/serum components during MH surgery with or without a retinal detachment improved the hole closure rate by facilitating the wound healing process [9]. Therefore, we suggest that a blood clot accumulated around the residual posterior vitreous membrane facilitated the MH closure and then the retinal detachment resolution. Another possible cause is that the release of vitreous traction, some of the subretinal fluid may have escaped, and the retinal pigment epithelium may have pumped out the subretinal fluid to help promote rapid attachment due to the lack of macular traction.

**Table 1:** Comparisons between the findings of previous studies and our study in eyes with pathologic myopia that had a natural resolution of macular hole retinal detachment

Publication, Year	Age	Axial Length (mm)	ERM	Posterior Staphyloma	Myopic Maculopathy	MH size (μm)	Mechanism of Spontaneous Resolution of MHRD	Time to reattachment
Yu J [6], 2014	64	31.4	–	+	Diffuse atrophy	Small (66)	MH closure and SRF absorption	45 months
Lee SJ [5], 2017	73	28.6	+	+	CNV related macular atrophy	Large (unknown)	Release of vitreo-retinal traction force	24 months
Tam SM [4], 2000	65	30.4	+	+	CNV related macular atrophy	Large (200)	Release of vitreo-retinal traction force	1 month
Our Case	76	31.0	+	+	Diffuse atrophy, Inflammatory CNV	Small (80)	MH closure and SRF absorption, blood clot	5 days

*MHRD* macular hole retinal detachment, *ERM* epi-retinal membrane, *MH* macular hole, *SRF* sub-retinal fluid, *CNV* choroidal neovascularization

We describe our findings of a rare case of pathologic myopia where an active CNV may have triggered a MHRD followed by a rapid and spontaneous resolution of both conditions in 5 days. Future detailed and long-term investigations using multimodal imaging on such inflammatory conditions will be required to determine the pathogenesis of the MHRD in eyes with pathologic myopia.

## Data Availability

All data generated or analyzed during this study are included in this published article.

## Abbreviations

CNV: Choroidal neovascularization
MHRD: Macular hole retinal detachment
SS-OCT: Swept-source OCT
FA: Fluorescein angiography
anti-VEGF: Anti-vascular endothelial growth factor
PIC: Punctate inner choroidopathy
MFC: Multifocal choroiditis
RPE: Retinal pigment epithelium
ERM: Epi-retinal membrane
MH: Macular hole
SRF: Sub-retinal fluid
